# Immunophenotype Expressions and Cytokine Profiles of Influenza A H1N1 Virus Infection in Pediatric Patients in 2009

**DOI:** 10.1155/2014/195453

**Published:** 2014-02-18

**Authors:** Shih-Min Wang, Yu-Ting Liao, Yu-Shiang Hu, Tzong-Shiann Ho, Ching-Fen Shen, Jen-Ren Wang, Yee-Shin Lin, Ching-Chuan Liu

**Affiliations:** ^1^Department of Emergency Medicine, College of Medicine, National Cheng Kung University and Hospital, Tainan 70428, Taiwan; ^2^Center of Infectious Disease and Signaling Research, National Cheng Kung University, Tainan 70428, Taiwan; ^3^Department of Pediatrics, College of Medicine, National Cheng Kung University and Hospital, Tainan 70428, Taiwan; ^4^Department of Medical Laboratory Science and Biotechnology, College of Medicine, National Cheng Kung University, Tainan 70428, Taiwan; ^5^Department of Microbiology & Immunology, College of Medicine, National Cheng Kung University, Tainan 70428, Taiwan

## Abstract

*Background*. A novel swine-origin influenza A H1N1 virus (S-OIV) caused human infection and acute respiratory illness in 2009, resulting in an influenza pandemic. *Objectives*. This study characterized the immune responses of S-OIV infection in pediatric patients at risk of pulmonary complications. *Methods*. All enrolled pediatric patients were confirmed virologically for S-OIV infection in 2009-2010, prospectively. Changes in cellular immunophenotypes were analyzed using flow cytometry. Plasma cytokine levels associated with S-OIV infection by pulmonary and without pulmonary complications were measured using cytokine cytometric bead assay kits. *Results*. A total of 85 patients with a mean age of 10.3 years were recruited. The level of C-reactive protein (CRP) was high in patients exhibiting pulmonary complications. The percentage of cellular immunophenotypes did not change between patients with and without pulmonary complications. The absolute numbers of peripheral blood mononuclear cells (PBMC), CD3, CD8, and CD16CD56 decreased with acute S-OIV pulmonary complications. Acute influenza infection with pulmonary complications was associated with high plasma concentrations of IL-1**β**, IL-6, IL-12, and IFN-**γ**. *Conclusion*. Immunophenotype studies have reported variability in immune response to the severity of S-OIV infections. Acute phase cytokine profiles of the 2009 S-OIV infection might have contributed to the pathogenesis of the pulmonary complications.

## 1. Introduction

Influenza pandemics have occurred at irregular intervals in the past, and supposing that another “new” influenza pandemic would occur in the future is justified. Influenza viruses cause seasonal epidemics because of the acquisition of mutations in the viral surface glycoproteins. Children have been disproportionately affected by pandemic influenza A H1N1, compared with older age groups. This infection has caused severe disease and death in few children [1].

H1N1 swine-origin influenza A virus (S-OIV) is a pandemic acute respiratory illness caused by a novel influenza H1N1 strain that first emerged in humans in Mexico and the United States in March 2009 and early April 2009. The cumulative total of cases reported from various regional offices of the World Health Organization (WHO) as of September 13, 2009, was more than 296 471, including at least 3486 deaths; the number that the WHO acknowledges understates the actual numbers [2]. A preliminary analysis of S-OIV proteins involved in viral virulence and pathogenicity revealed that they are highly similar to strains that cause mild symptoms in humans. In concordance, low mortality is observed outside Mexico, and the virus is unlikely to cause severe infections similar to those caused by the 1918 pandemic influenza or the H5N1 influenza [3]. However, the 2009 H1N1 influenza pandemic was associated with pediatric death rates that were 10 times the rates and double the hospitalization rates for seasonal influenza in previous years in Argentina [4].

Influenza A virus-infected epithelial cells and leukocytes respond to the infection by producing chemokines, proinflammatory, and regulatory cytokines. Cytokine responses to influenza viral infection contribute to the pathogenesis and severity of influenza viral infection. A *cytokine storm* contributes to the bronchiolitis and alveolar edema characteristics of severe influenza pneumonia, and at least some of these inflammatory mediators are produced by virus-infected lung epithelium [5]. Several independent studies have documented an early rise in TNF-*α*, IL-1 *α* and *β*, and IL-6 in bronchoalveolar lavage (BAL) fluids in temporal association with clinical symptom and lung pathology [6].

Immune responses to viral infections involve a complex orchestration between innate signals and adaptive responses of specific T and B cells. Viral infections are known to predominantly induce Th1 immunity that promotes the activation of CD8 T cells and macrophage functions and causes B cell differentiation. Identification of early immunological parameters is critical for the study of the pathogenesis of this disease [7]. Substantially limited evidence has been reported on host T cell responses to the pandemic H1N1 S-OIV infection in humans. This study characterized cytokine response and immunophenotype expressions during the 2009 H1N1 viral infection in pediatric patients with lower respiratory tract infection and included patients with upper respiratory tract infection as a comparative group.

## 2. Materials and Methods

### 2.1. Patient Enrollment

All 24 patients with S-OIV infection pulmonary complication and 61 patients with S-OIV upper respiratory tract infection were studied. All of the patients presented at the Departments of Emergency Medicine and Pediatrics of National Cheng Kung University Hospital (NCKUH) between August 2009 and January 2010. Inclusion criteria were age below 18 years, presenting with acute febrile respiratory illness, and confirmed virologically for S-OIV infection. Informed consent was obtained from each participating patient or the patient's parents or guardian. This study was approved by the Institutional Review Board of NCKUH.

### 2.2. Case Definition

A confirmed case of pandemic influenza A (H1N1) viral infection was defined by a nasopharyngeal swab positive for S-OIV by viral culture or RT-PCR. Patients were stratified into 2 categories based on clinical features and radiological findings: (1) upper respiratory tract infections, diagnosed with rhinitis, pharyngitis, or otitis media which were present without signs of lower respiratory tract infection and (2) lower respiratory tract infections, including bronchitis, bronchiolitis, bronchopneumonia, and pneumonia, diagnosed with signs of lower airway involvement (tachypnea, dyspnea, retraction, wheezing, or rales) or infiltrates revealed by a chest X-ray.

### 2.3. Virological Studies

Pharyngeal swabs were collected from all recruited patients and were submitted to the Virology Laboratory in NCKUH for investigation. The protocol of the US Centers for Disease Control and Prevention of real-time RT-PCR for S-OIV 2009, as recommended by the WHO, was used. The PCR products were sequenced for further confirmation by using a standard high-throughput sequencing system of the BigDye Terminator, version 3.1 (Applied Biosystems, Foster City, CA, USA) featuring a 1-mm^3^ double-stranded template.

### 2.4. Flow Cytometric Analysis

Peripheral blood mononuclear cells (PBMCs) were isolated from ethylenediamine tetraacetic acid (EDTA) whole blood by Ficoll separation (Ficoll-Paque plus; Amersham Biosciences). Whole blood (150 *μ*L) was incubated on ice by using 10 *μ*L of each antibody for 15 minutes in the dark. Two milliliters of FACS lysing solution (Becton Dickinson) was added and incubated at room temperature for 10 minutes. The cells were then washed using PBS and fixed using 0.5 mL of 0.1% glutaraldehyde solution in PBS. Stained lymphocytes were analyzed using flow cytometry (Becton Dickinson Immunocytometry Systems). Data were acquired and analyzed using Cell Quest software (Becton Dickinson). The following fluorescent MAbs were used: peridinin chlorophyll protein-conjugated Leu 4 (CD3; pan T), phycoerythrin-conjugated Leu-3a (CD4 T cells), Leu-2a (CD8 T cells and NK cells), Leu-11c (CD16; NK lymphocytes), and Leu-19 (CD56; natural killer (NK) lymphocytes and T lymphocyte subset). IgG1 isotype control antibody conjugates were included in all assays to determine background fluorescence.

### 2.5. Measurement of Cytokines

EDTA blood samples were immersed in ice and immediately transported to the laboratory for processing. Plasma was separated by centrifugation (2000 g for 10 min) at 4°C and stored in 300 *μ*L aliquots at −70°C until analysis. The cytometric bead array assay (CBA) (BD Pharmingen, CA, USA) consisted of six bead populations exhibiting distinct fluorescence intensities. The concentrations of IL-1*β*, IL-6, IL-8, IL-10, IL-12, and IFN-*γ* were measured using the human Th1/Th2 cytokine CBA kits.

### 2.6. Statistical Analysis

Data were expressed as mean ± SD. Statistical significance was determined by conducting paired or nonpaired nonparametric tests and using SPSS (version 18.0; Chicago, IL, USA). The nonparametric Mann-Whitney test was used to compare cytokine concentrations between influenza-infected patients. *P* < 0.05 was considered significant.

## 3. Results

### 3.1. Participant Characteristics

A total of 85 cases of children and adolescents infected with influenza visited our hospital during the 2009 and 2010 influenza seasons. Virologically confirmed case-patients exhibited a median age of 10.3 years (range, 2 months–17.7 years) and 47% were males.  Table 1 shows the demographic data of pediatric patients infected with pandemic S-OIV and presenting with or without pneumonia. No variation in age, gender, hospitalization rate, and length of stay between the groups were observed. No patients had underlying diseases or complicated with acute respiratory distress syndrome (ARDS) after infection.

### 3.2. Laboratory Tests on Admission

The hemogram of WBC, hemoglobin level, and platelet count were similar in patients who presented with or without pulmonary complications. Conversely, children presenting with pandemic S-OIV pneumonia were more likely to have a high level of CRP (19.7 ± 28.9 mg/L versus 8.0 ± 8.4 mg/L, *P* = 0.04). High levels of aspartate aminotransferase (AST) (56.2 ± 76.9 U/L versus 33.4 ± 9.8 U/L, *P* = 0.03), alanine aminotransferase (ALT) (22.2 ± 45.7 U/L versus 13.6 ± 5.1 U/L, *P* < 0.01), and creatine kinase (CK) (229.2 ± 467.4 U/L versus 157.8 ± 178 U/L, *P* < 0.01), but not of alkaline phosphatase (ALK-P), were detected in patients who presented without pulmonary complications (Table 1).

### 3.3. Immunophenotype Analysis

The absolute neutrophil count, absolute monocyte count, and absolute lymphocyte count in S-OIV infected patients presenting with pulmonary complications did not considerably differ from those who presented without pulmonary complications; however, a difference in immunophenotypes after further analysis was observed. To determine the lymphocyte and NK cell expression of S-OIV infection, the activation of CD3, CD4, CD8, CD20, and CD16CD56 was evaluated (Table 2). Acute S-OIV infection along with pulmonary complication was associated with significantly low absolute numbers of CD3 (244 ± 188 cell/mm^3^ versus 478 ± 386 cell/mm^3^, *P* = 0.02), CD8 (119 ± 85 cell/mm^3^ versus 239 ± 179 cell/mm^3^, *P* = 0.02), and CD16CD56 (63 ± 41 cell/mm^3^ versus 130 ± 100 cell/mm^3^, *P* = 0.04). The ratio of CD4/CD8 (0.9 ± 0.3 versus 1.02 ± 0.35, *P* = 0.51) did not change markedly between the groups.

### 3.4. Cytokine Expression

Patients presenting with pulmonary complications exhibited significantly higher concentrations of IL-1*β*, IL-6, IL-12, and IFN-*γ* compared with those who presented without pulmonary complications. No differences in IL-8 (88.54 ± 123.55 pg/mL versus 29.57 ± 21.76 pg/mL) and IL-10 (5.7 ± 4.63 pg/mL versus 5.43 ± 8.05 pg/mL) levels were observed between patients presenting with and without pulmonary complications (Table 3).

## 4. Discussion

An inflammatory response of the host to the influenza virus occurs in infection with the release of pro- and anti-inflammatory cytokines. Although cytokine production is necessary for the defense function, an excessive response can cause deleterious effects. IL-6 has been suggested to be an early inflammatory marker, and levels correlate well with the severity and prognosis of sepsis. Systemic inflammation related to lung infections is generally caused by cytokine translocation from the lungs into the systemic circulation [7]. In addition, systemic increase in IL-6 and IL-10 in pneumonia is associated with the worse prognosis [8]. However, we did not detect the substantially raised level of IL-10 in patients presenting with or without complication. This finding may be related to host inflammatory response, which is an evolutionary process over time that varies depending on the number of days and several other factors, such as the use of antiviral agents for treatment of and vaccination for prophylaxis of influenza infection [9].

IL-1 is a potent proinflammatory cytokine that is essential for host-pathogen response. IL-1*β* plays a crucial role in acute and chronic lung inflammatory diseases [10]. Chiaretti and colleagues reported that IL-1*β* and IL-6 expressions in plasma were considerably upregulated in H1N1 infected patients presenting with severe diseases and correlated with the severity of respiratory compromise and fever [11]. In this study, we performed a comparative study by pulmonary complication. The cytokine expression did not indicate a correlation with hypoxemia or extent of lung infiltrates. The IL-1*β* activity increased in bronchoalveolar lavage fluid of patients presenting with ARDS. A microarray analysis on ARDS-associated pulmonary edema indicated a prominent role for IL-1*β* [12]. Influenza A virus-infected monocytes and macrophages efficiently produce large quantities of IL-1*β* and TNF-*α* [13]. IL-1*β* may enhance TNF-*α*-induced neutrophil recruitment to the lung by altering TNF receptors as well as MIP-2 and KC production in pulmonary diseases [14]. This supports the notion that slightly high plasma levels of IL-1*β* are exhibited in patients presenting with pulmonary complications.

IL-12 is a heterodimeric cytokine which was primarily produced by macrophages and dendritic cells in both innate and adaptive immune responses. Plasma levels of IL-12p40 were considerably high in the H5N1-positive group. IL-12p40 may be released from alveolar epithelial or endothelial cells to circulate in the blood, suggesting the contribution of macrophages to lung injury at the initial stage of H5N1 infection [15]. Dendritic cells have been observed to produce relatively high levels of IL-12 in response to influenza A viral infection or dsRNA stimulation [16, 17]. IL-12 exerted a direct effect on IFN regulatory factor-1 (IRF-1), an effect directly mediated by the induction of IFN-*γ* production [18]. However, Matsumoto and coworkers observed that serum concentrations of IFN-*γ* were substantially low in H1N1 pneumonia patients presenting with neutrophilic leukocytosis [19]. IFN-*γ*-mediated enhancement of IL-12 production also represented the convergence of signals derived from innate and adaptive immune responses [20]. These findings may support the notion that plasma levels of IL-12 and IFN-*γ* were elevated in patients presenting with pulmonary complications.

The T cell immune response is characterized by expansion of naive CD4 and CD8 cells into effector T cells specific for some influenza viral proteins. CD4 and CD8 T cell response against the influenza virus have most often been described in animal models. Eliminating the H3N2 virus is substantially delayed in animal deficient of CD8 cells [21]. CD8 cell clonal expansion associated with the primary or secondary challenge is a crucial determining factor in controlling influenza infection [22]. Giamarellos-Bourboulis et al. [23] demonstrated that considerably few CD4 positive T cells and B cells were present in critically ill patients. These findings may provide partial explanations for the decrease in CD3 and CD8 T cells in inpatients presenting with pulmonary complications.

NK cells are increased in the lungs following influenza infection [24]. NK cells help CD8^+^ cell function indirectly by secreting soluble factors [25] or by stimulating dendritic cells to produce IL-12 [26]. Peripheral blood of CD16CD56 cells was decreased in patients presenting with pulmonary complications, which might reflect homing of these cells to the respiratory tract [27] or increased apoptosis and activation-induced cell death [28]. Furthermore, influenza can infect NK cells, thereby triggering NK cell death and potentially providing a mechanism for the virus to escape innate immune defenses [29]. NK cells considerably augment pulmonary inflammation, contributing to the pathogenesis of influenza infection [30].

The 2009 S-OIV appears to be highly infectious but exhibits a relatively low mortality for a pandemic strain. An antibody response can prevent infection with the T cell response limiting morbidity. These data contribute to a better understanding of the innate and adaptive immune mechanisms at play during acute influenza infection in pediatric patients, providing a basis for further analysis into the contribution of these inflammatory mediators and cells in controlling human influenza infection.

## Figures and Tables

**Table 1:** Demographic and laboratory findings in children infected with 2009 H1N1.

	Without pulmonary complication (*N* = 61)	With pulmonary complication (*N* = 24)	*P* value*
Age (years)	9.8 ± 4.3	10.8 ± 4.1	0.33
Male (%)	30 (73.1%)	10 (50%)	0.14
Hospitalization (%)	9 (21.9%)	4 (20%)	0.87
LOH (days)	4.1 ± 1.9	3.8 ± 0.5	0.77
WBC (×10^3^/cmm), (*n*)	6.2 ± 1.9 (40)	6.9 ± 3.6 (20)	0.88
ANC (/cmm)	4733 ± 2141	4904 ± 3062	0.94
AMC (/cmm)	501 ± 254	520 ± 321	0.49
ALC (/cmm)	917 ± 606	1036 ± 928	0.84
Hemoglobin (g/dL)	13.3 ± 1.3	13.4 ± 1.1	0.74
Platelet (×10^3^/cmm)	219 ± 58	218 ± 57	
CRP (mg/L)	8.0 ± 8.4 (38)	19.7 ± 28.9 (18)	0.04
AST (U/L)	56.2 ± 76.9 (34)	33.4 ± 9.8 (19)	0.03
ALT (U/L)	22.2 ± 45.7 (35)	13.6 ± 5.1 (19)	<0.01
CK (U/L)	229.2 ± 467.4 (34)	157.8 ± 178 (16)	<0.01
ALK-P (U/L)	226.7 ± 74.8 (26)	205.2 ± 82.2 (13)	0.56

ALC: absolute lymphocyte count; ALK-P: alkaline phosphatase; ALT: alanine aminotransferase; AMC: absolute monocyte count; ANC: absolute neutrophil count; AST: aspartate aminotransferase; CK: creatine kinase; CRP: C-reactive protein; LOH: length of hospitalization; WBC: white blood cell.

*Chi-square test or Mann-Whitney test.

**Table 2:** The absolute cell number of immunophenotypes in patients infected with 2009 H1N1 according to pulmonary complications.

Cell markers (cell/mm^3^)	Without pulmonary complication (*N* = 24)	With pulmonary complication (*N* = 15)	*P* value
CD3	478 ± 386	244 ± 188	0.02
CD4	222 ± 206	122 ± 95	0.1
CD8	239 ± 179	119 ± 85	0.02
CD20	57 ± 42	47 ± 37	0.48
CD16CD56	130 ± 100	63 ± 41	0.04
CD14	94 ± 70	142 ± 158	0.95
CD4CD25	13 ± 13	6 ± 4	0.22

**Table 3:** The cytokines profile of patients infected with 2009 novel H1N1 according to pulmonary complications.

Cytokine (pg/mL)	Without pulmonary complication (*N* = 34)	With pulmonary complication (*N* = 24)	*P* value
IL-1*β*	1.7 ± 1.2	3.1 ± 1.9	0.01
IL-6	10.5 ± 7.7	21.5 ± 18.9	<0.01
IL-8	29.6 ± 21.8	88.5 ± 123.6	0.09
IL-10	5.4 ± 8.0	5.7 ± 4.6	0.53
IL-12	1.5 ± 4.0	2.2 ± 0.8	<0.01
IFN-*γ*	1.4 ± 1.1	2.8 ± 0.9	<0.01

IFN: interferon; IL: interleukin.
